# 

**Published:** 1897-12

**Authors:** 


DECEMBER, 1897.

THE

### AMERICAN JOURNAL

OF

### DENTAL SCIENCE.

LIBRARY
SURGEON GENERAL'S OFFICE
DEC.-27.-1897

EDITED BY

F. J. S. GORGAS, M. D., D. D. S.
RICHARD GRADY, M. D., D. D. S.

Vol. 31. THIRD SERIES No. 8.

PUBLISHED MONTHLY.

$2.50 PER ANNUM.

BALTIMORE:
SNOWDEN & COWMAN MFG. CO., 9 W. FAYETTE ST.

LONDON:
TRUBNER & CO., 60 PATERNOSTER ROW.

Entered at the Post Office at Baltimore, Md., as Second-class Matter.

PAYABLE IN ADVANCE.

